# Seroprevalence and risk factors of antibodies against *Coxiella burnetii* among dog owners in southwestern Québec, Canada

**DOI:** 10.1017/S0950268821001412

**Published:** 2021-06-28

**Authors:** L. Duplaix, P. Turgeon, B. Lévesque, J.-P. Rocheleau, A. Leboeuf, I. Picard, K. Manguiat, H. Wood, J. Arsenault

**Affiliations:** 1 Department of Pathology and Microbiology, Faculty of Veterinary Medicine, Université de Montréal, Saint-Hyacinthe, Québec, Canada; 2 Groupe de recherche en épidémiologie des zoonoses et santé publique (GREZOSP), Faculty of Veterinary Medicine, Université de Montréal, Saint-Hyacinthe, Québec, Canada; 3 National Microbiology Laboratory, Public Health Agency of Canada (PHAC), Saint-Hyacinthe, Québec, Canada; 4 Département de médecine sociale et préventive, Faculté de médecine, Université Laval, Québec, Canada; 5 Cégep de Saint-Hyacinthe, Saint-Hyacinthe, Québec, Canada; 6 Ministère de l'Agriculture, des Pêcheries et de l'Alimentation du Québec (MAPAQ), ville de Québec, Québec, Canada; 7 National Microbiology Laboratory, Public Health Agency of Canada (PHAC), Winnipeg, Manitoba, Canada

**Keywords:** Coxiellae, epidemiology, Q fever, serology

## Abstract

*Coxiella burnetii* is a zoonotic agent responsible for human Q fever, a potentially severe disease that can lead to persistent infection. This cross-sectional study aimed to estimate the seroprevalence to *C. burnetii* antibodies and its association with potential risk factors in the human population of five regions of Québec, Canada. A serum bank comprising sera from 474 dog owners was screened by an enzyme-linked immunosorbent assay followed by confirmation of positive or equivocal sera by an indirect immunofluorescence assay. Observed seroprevalences of 1.2% (95% confidence interval (CI): 0.0–6.6), 2.6% (95% CI: 0.5–7.4) and 5.9% (95% CI: 3.4–9.6) were estimated in the regions of Montréal, Lanaudière and Montérégie, respectively, which all included at least 83 samples. Having lived or worked on a small ruminant farm (prevalence odds ratio (POR) = 5.4; 95% CI: 1.6–17.7) and being a veterinarian or veterinary student (POR = 6.1; 95% CI: 1.6–24.0) were significantly associated with *C. burnetii* seropositivity. Antibodies against *C. burnetii* were detected in the human population of Québec. Although seropositivity to this agent was associated with occupational contact with domestic animals, antibodies were also detected in people with no reported professional exposure. No associations with ruminant farm proximity were identified.

## Introduction

The zoonotic bacterium *Coxiella burnetii* is the causative agent of Q fever in humans [1]. A variety of animal species can get infected with *C. burnetii*; however, the main sources of human infection with this pathogen are domestic ruminant populations and infection with this bacterium mainly occurs through inhalation of contaminated aerosols [1, 2]. In humans, primary *C. burnetii* infections are predominantly asymptomatic, but acute illness may occur and the infection might also become persistent [1, 2]. Although persistent *C. burnetii* infections occur in <5% of primary infections, the most likely associated outcomes (endocarditis and vascular infection) can lead to death [1].

Many large Q fever outbreaks have been described in Europe in the literature, which were generally associated with living in proximity to an infected small ruminant farm or in a region with high goat density [3, 4]. In urban areas, a few outbreaks were also suspected to have been caused by contact with parturient cats or dogs [5–7] or linked to the dispersion of contaminated hay, manure and dust by a farm truck passing through an urban zone [8]. However, Q fever outbreaks remain rare epidemiological events, whereas endemic cases are reported on a regular basis in many countries including Canada. Population-based regional studies had reported seroprevalence estimates for *C. burnetii* ranging from 2.4% to 12.8% in various countries, suggesting a significant risk of contracting the infection [9]. In areas where *C. burnetii* infection is endemic in the cattle population, it has been reported that the general population is at risk of contracting the infection, even for those without contact with ruminants [10]. Due to its nonspecific clinical manifestations, the disease is likely underdiagnosed and thus left untreated. This is concerning as treatment of acute cases is recommended to shorten the illness and reduce the risk of severe complications [1, 11]. Therefore, it is relevant that clinicians recognise those at a higher risk of *C. burnetii* seropositivity to ensure they are tested, diagnosed, properly followed and treated if necessary. Although professional exposure to animals has been identified as a risk factor for Q fever, such as working with cattle [12], only little information is available in the literature on the risk factors associated with endemic Q fever in the general population, especially in urban areas. Such information is essential to guide diagnostic, prevention and control measures.

Q fever is a notifiable disease in the province of Québec, Canada [13], with an annual incidence rate of 0.4 reported cases per 100 000 person-years in 2017 [14]. In Canada, whereas high *C. burnetii* seroprevalences were estimated among individuals in close contact with animals, such as small ruminant veterinarians and veterinary students (59%) of Ontario, and trappers (15%) and shepherds (28.4%) of Québec [15–17], little is known about the risk distribution in the general population. Therefore, this study aimed to estimate *C. burnetii* seroprevalence and associated risk factors in humans living in southwestern Québec.

## Materials and methods

### Study design and selection of the participants

This study used the sera bank and questionnaire data from a parent cross-sectional study about environmental risk factors for seropositivity to arboviruses in dogs and humans conducted in 2014, in combination with questionnaire data collected in 2018 from a sub-sample of the participants of the parent study. The parent study was conducted in five administrative regions of southern Québec where arbovirus activity was reported: Montréal, Laval, Montérégie and the southern part of Lanaudière and Laurentides (see Table 1 for a description of the regional characteristics). The parent study used a convenience sampling method to recruit dogs at 89 randomly selected veterinary clinics or hospitals from a provincial registry. Dog owners from the 1442 recruited dogs were consecutively contacted until 485 (one or more people of at least 18-year-old living at the same address as the dog) consented to be sampled with the goal to estimate the prevalence of arbovirus infections [18]. Following consent, blood samples were collected by a nurse from the 485 participants (367 households) at their homes between 27 March 2014 and 10 June 2014. Participants were asked to answer a socio-demographic and behavioural questionnaire at the time of sampling [18]. Blood samples were centrifuged upon collection and sera were kept frozen at −80 °C.

**Table 1.** Regional characteristics of five administrative regions of southwestern Québec, Canada

Demographic characteristics	Administrative region				
	Lanaudière	Laurentides	Laval	Montréal	Montérégie
Total population as of 1 July 2014^a^	492 234	586 051	420 870	1 988 243	1 508 127
% of Québec population in 2014^a^	6.0	7.1	5.1	24.2	18.4
Cattle production^b^					
Number of farms	519	538	2	2	2733
Min-max (mean) of animal/farm	1-2036 (1019)	1-1820 (911)	80-124 (102)	5-158 (82)	1-2280 (1141)
Total number of animals	37 455	41 732	204	163	189 083
Goat production^b^					
Number of farms	35	56	2	1	164
Min-max (mean) of animal/farm	1-200 (101)	1-120 (61)	7-60 (34)	5	1-388 (195)
Total number of animals	716	632	67	5	7 056
Sheep production^b^					
Number of farms	91	80	3	1	279
Min-max (mean) of animal/farm	2-1250 (626)	1-645 (323)	4-40 (22)	7	1-1725 (863)
Total number of animals	13 127	7 551	54	7	32 669
Area (km^2^) in 2011^a^	12 422	20 771	247	499	11 131

a Data obtained from the Institut de la statistique du Québec.

b Calculated from data provided by the MAPAQ; it comprises all bovine, caprine and ovine of all production type (dairy and meat) that were registered in 2010 and 2014. Averages for 2010 and 2014 are presented.

During the Summer 2018, the 485 participants of the parent study were recontacted and invited to participate in the current study. Their participation entailed the use of their previously collected serum and questionnaire data as well as answering, on a voluntary basis, a new questionnaire on their risk of exposure to *C. burnetii*. The available sample of three (Lanaudière, Montérégie and Montréal) out of the five administrative regions was sufficient (⩾19 participants) to estimate the prevalence of *C. burnetii* with a precision of 0.05 assuming a confidence level of 95% and an expected *C. burnetii* seroprevalence of 1.2% as reported in controls in a previous study realised in Québec [15], as calculated in Epitools [19]. A power analysis was conducted to evaluate which strength of association between possible risk factors and the presence of antibodies against *C. burnetii* could be detected assuming the overall prevalence obtained with the prevalence study.

### Questionnaires

The parent study questionnaire was used to gather data on the socio-demographic characteristics of participants, such as their sex, age, profession, number of hours per week spent outdoors in summer and place of residence in the last 10 years (from 2004 to 2014), as well as their full home address.

The 2018 Q fever questionnaire asked participants to refer back to the year 2014 (year of sample collection) or up to 5 years prior to sampling (i.e. 2009–2014) when answering the questions, since several studies have reported the persistence of detectable levels of *C. burnetii* antibodies for at least 5 years [20]. Therefore, a 5 year-retrospective period prior to sampling was selected to not only consider the persistence of antibodies but also to limit poor recall. The Q fever questionnaire included questions on: (1) contact with domestic ruminants (dairy or meat cattle, goat, sheep and cervids), gestating dogs or cats and new-born kittens and puppies during work and/or leisure activities; (2) hunting or trapping activities and (3) raw milk consumption. This questionnaire was pretested by three researchers and two members of the general population in May 2018 for feedback on clarity of content and time of completion. Participants were invited to complete the Q fever questionnaire online using a survey platform (SurveyMonkey).

### Density of and proximity to domestic ruminants

The geographical coordinates and the size (e.g. number of bovine, caprine and ovine heads) of each registered farm in Québec for the years 2010 and 2014 were obtained from the Ministère de l'Agriculture, des Pêcheries et de l'Alimentation du Québec (MAPAQ). The average number of animals of each species in each farm was calculated for 2010 and 2014. The 2014 participants' home addresses were geo-referenced using their house number, street name and six-digit postal code using GeoPinpoint Suite 6.4 software (DMTI Spatial Inc., ON, Canada). The distance between each participants' 2014 home address and the nearest cattle, sheep and goat farm was estimated in ArcGIS version 10.5.1 (ESRI, Redlands, CA, USA), as well as the number of farms and animals in a 5 km radius around the place of residence of each participant. This distance was chosen based on several studies conducted during outbreaks where living within a 5 km radius of a ruminant farm infected with *C. burnetii* was associated with increased incidence of infection or prevalence of antibodies against *C. burnetii* in humans [21–23].

### Serological tests

The sera were screened for the presence of immunoglobulin G (IgG) antibodies to *C. burnetii* phase II antigen using the Panbio *Coxiella burnetii* (Q fever) IgG ELISA kit (Alere Inc., Ottawa, Ontario). The Panbio IgG ELISA kit has been reported to have a sensitivity of 71% and a specificity of 96% [24]. Serum samples with a positive or equivocal result (Panbio index values ⩾ 0.9) or with a negative result that was close to the threshold (Panbio index value = 0.8) after screening with the enzyme-linked immunosorbent assay (ELISA) were confirmed using an immunofluorescence assay (IFA) for the detection of IgG antibodies against phase I and phase II *C. burnetii* antigens (Focus Diagnostics, USA). IFA positivity was determined using a cut-off titre of 1:32 to either phase I or phase II IgG antibodies. All tests were performed at the National Microbiology Laboratory of the Public Health Agency of Canada (NML) in Winnipeg, Canada, according to the manufacturers' instructions.

### Statistical analysis

The seroprevalences of *C. burnetii* with exact 95% confidence intervals (CIs) were estimated for each region with adjustment for household clustering.

The associations between the explanatory variables extracted from the questionnaires and from the animal production database and *C. burnetii* seropositivity were estimated using logistic regression models. A sub-analysis was performed on participants who had not moved in the 10 years prior to blood collection to investigate the association between living in proximity to ruminant farms and *C. burnetii* seropositivity. Continuous explanatory variables were categorised intro three groups: those with a value of 0, and, among those larger than 0, below and above the median. Categories showing few or no observations were combined for categorical variables to ensure model convergence. Univariable analyses were performed and variables with associations with a *P* value <0.20 were selected for inclusion in a multivariable model. In the presence of two strongly correlated variables (prevalence odds ratio (POR) >8), only one variable was retained based on higher biological relevance or smaller *P* value; if they were very similar for both criteria, alternative models were built. A manual backward selection was used to determine the final model using a *P* value >0.05 as a criterion for rejection. However, when the removal of a variable changed the estimated PORs of other statistically significant variables present in the model by more than 20%, it was retained in the model as a potential confounder. The fit of the final model was evaluated with the Hosmer–Lemeshow goodness-of-fit test. A sensitivity analysis was performed to assess the possible effect of household clustering by randomly selecting one participant per household and re-estimating the final multivariable model; this procedure was repeated 10 times. Similar associations were obtained, and all predictors remained statistically significant, suggesting a negligible household clustering effect. Therefore, the full sample was used for the final multivariable model. POR with 95% CIs were used to present the results. The available sample size with complete information (i.e. 316 participants including 17 with seropositive results) allows for the detection of POR of ⩾4 for a dichotomous risk factor (in which at least 20% of participants were included in each category) at a statistical power of 80% and alpha of 5%. These analyses were conducted using SAS version 9.4 (SAS Institute Inc., Cary, NC, USA).

The spatial distribution of each participant with their serological result, and the bovine and the small ruminant farm densities in a 5 km radius were mapped using ArcGIS. The presence of high and low risk circular clusters of seropositivity was explored using the Kulldorff spatial scan statistic [25] performed in SatScan version 9.7 software (MA, USA). A Bernoulli model was used with cases and controls defined as seropositive and seronegative participants, respectively, with a maximum cluster size representing 50% of the study sample and a minimum number of two cases per cluster. Two models were performed, one with all participants and the other restricted to participants having reported no occupational contact with animals. Statistical significance (alpha = 0.05) was determined using 9999 Monte Carlo replications.

## Results

### Study population

**Fig. 1.**
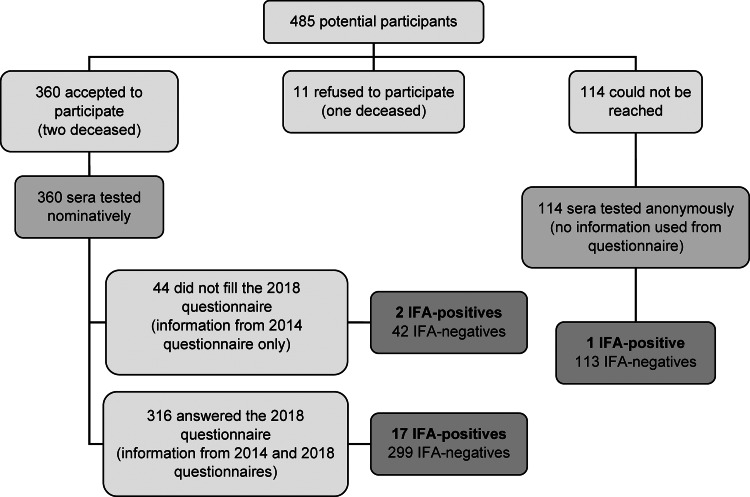
Flowchart illustrating the selection of participants, collection of information and results for a *C. burnetii* seroprevalence study in Québec, Canada, from a source population of 485 potential participants from which sera were available from a 2014 study.

Nearly three quarters of the participants in the parent study on arboviruses – 358 individuals and the family members of two deceased participants – agreed to take part in the current study on *C. burnetii*. Ten people refused to participate and a family member from one deceased participant could not be reached. The other 114 potential participants could not be reached (absence of reply or invalid contact information). Sera from the latter individuals were tested anonymously and included in the prevalence estimation per region (Fig. 1).

The Q fever questionnaire was fully completed by 308 (86% of those successfully contacted and consenting) participants; partial responses were obtained from eight additional participants. These 316 participants were from 257 different households with one, two and three participants selected from 199, 57 and 1 household, respectively.

### Serological results

**Fig. 2.**
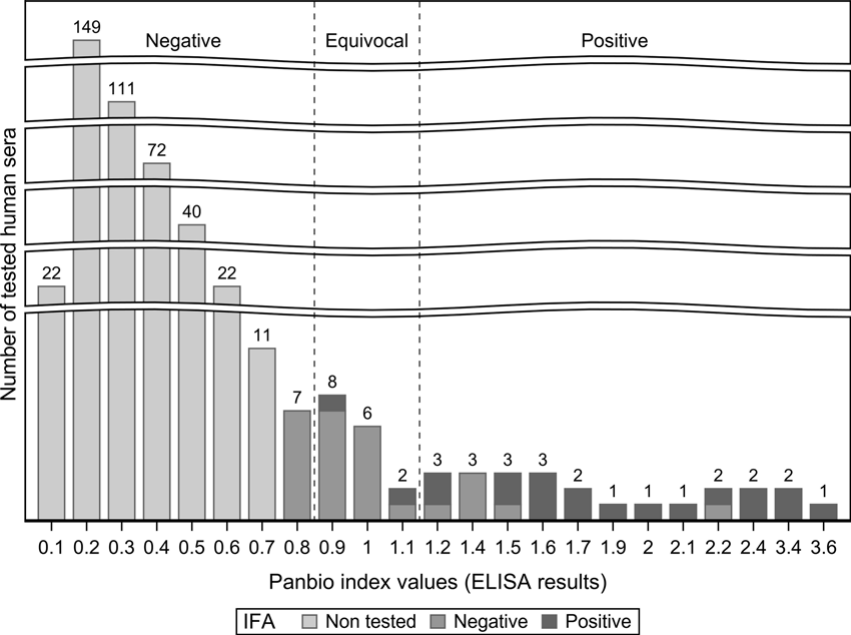
Distribution of human sera tested by ELISA for *C. burnetii* according to their Panbio index values (quantitative results) and their qualitative interpretation with the IFA results for the ELISA-positive sera, ELISA-equivocal sera and ELISA-negative sera with a Panbio index values close to the equivocal threshold. The sera were collected in human participants of Québec, Canada, in 2014 and tested in 2019.

Twenty-four and 16 sera of the 474 tested by ELISA were positive and equivocal, respectively, among which 20 (4.2% of the total sample) were IFA-positive (Fig. 2). The IgG antibody titres of both phases obtained by the IFA are presented in Table 2. The highest estimated seroprevalence was obtained for the region of Laval with 11.1% (95% CI: 0.3–48.2) followed by the regions of Montérégie, Lanaudière and Montréal with seroprevalence estimates of 5.9% (95% CI: 3.4–9.6), 2.6% (95% CI: 0.5–7.4) and 1.2% (95% CI: 0.0–6.6), respectively. The region of Laurentides had the lowest result with 0% (95% CI: 0.0–23.2) (Table 3).

**Table 2.** IgG antibody titres against *C. burnetii* phase I and II antigens obtained with the IFA for the samples confirmed positives (*n* = 20)

Seropositive sample	IgG antibodies	
	Phase I	Phase II
1	<1:32	1:64
2	<1:32	1:128
3	<1:32	1:32
4	1:64	1:512
5	1:512	1:256
6	<1:32	1:32
7	<1:32	1:64
8	<1:32	1:64
9	<1:32	1:32
10	1:32	1:128
11	<1:32	1:32
12	<1:32	1:128
13	<1:32	1:256
14	1:32	1:128
15	<1:32	1:128
16	<1:32	1:64
17	<1:32	1:64
18	<1:32	1:128
19	1:512	1:2048
20	<1:32	1:64

**Table 3.** Estimated seroprevalence to *C. burnetii* with exact 95% CIs adjusted for clustering by household in all tested human participants by administrative regions of Québec, Canada, 2014 (*n* = 474)

Region	Number of participants	Number of seropositive participants	Observed seroprevalence (%)	
			Estimate	95% CI

Lanaudière	115	3	2.6	0.5–7.4
Laurentides	14	0	0.0	0.0–23.2^a^
Laval	9	1	11.1	0.3–48.2
Montérégie	253	15	5.9	3.4–9.6
Montréal	83	1	1.2	0.0–6.6

a 95% CIs not adjusted for clustering by household.

### Risk factor analysis

From the data obtained via the two questionnaires (summarised in Tables 4 and 5), seven variables with a *P* < 0.20 in the univariable analysis were selected for multivariable analysis. The variables ‘having occupational contact with animals and/or having contact with animals during leisure activities’ and ‘having occupational contact with animals’, both between 2009 and 2014, were highly correlated and hence only the latter, which had the smallest *P* value, was kept in the final model. The variable ‘region’ was excluded because it was closely related to other variables related to ruminant production. The three variables measuring living or working on a bovine farm, a small ruminant (sheep and/or goat) farm or any type of ruminant (cattle and/or sheep and/or goat) farm during a lifetime were all correlated and hence, multivariable models were built using each of those variables. When the variable measuring working or living on a small ruminant farm during a lifetime was used, the only other statistically significant variable was an occupational contact with animals during the period of interest. Categories (being an animal health technician and having another occupational contact with animals) of the latter variable were not statistically different and were combined for the multivariable analysis to improve the precision of model coefficients.

**Table 4.** Descriptive statistics of participants' characteristics collected via a questionnaire completed in 2014 and *P* value from univariable logistic regression modelling the seropositivity to *C. burnetii* in five administrative regions of Québec, Canada (*n* = 360)

Characteristics of participants	Number of participants	Seropositive participant		*P* value^a^
		*N*	%	
Administrative region of residency^b^				0.13
Lanaudière	83	3	3.6	
Laurentides	9	0	0.0	
Laval	6	1	16.7	
Montérégie	197	14	7.1	
Montréal	65	1	1.5	
Sex				0.33
Female	246	11	4.5	
Male	114	8	7.0	
Age^c^				0.95
18–24	19	0	0.0	
25–34	62	5	8.1	
35–44	87	4	4.6	
45–54	96	10	10.4	
55–64	65	0	0.0	
⩾65	31	0	0.0	
Number of hours spent outside per week in summer				0.66
⩽20	188	9	4.8	
>20	172	10	5.8	
Moved between 2004 and 2014				0.63
Yes	190	9	4.7	
No	170	10	5.9	

a Likelihood ratio test *P* value.

b The adjacent regions of Lanaudière and Laurentides were combined for analysis due to lack of model convergence secondary to category with no seropositive participants.

c The participants under the age of 44 as well as the participants over the age of 45 were respectively combined for analysis due to lack of model convergence secondary to categories with no seropositive participants.

**Table 5.** Descriptive statistics of participants' characteristics collected via a questionnaire completed in 2018 and *P* value from univariable logistic regression modelling the seropositivity to *C. burnetii* in five administrative regions of Québec, Canada (*n* = 316)

Characteristics of participants	Number of participants^a^	Seropositive participant		*P* value^b^
		*N*^a^	%	
Knowledge of Q fever/*C. burnetii*				0.26
Yes	43	4	9.3	
No	273	13	4.8	
Having lived or worked on a farm during a lifetime^c^				
*Any type of ruminant farm*				0.03
Yes	74	8	10.8	
No	242	9	3.7	
*Bovine farm*				0.06
Yes	68	7	10.3	
No	247	10	4.1	
*Small ruminant farm*				0.003
Yes	31	6	19.4	
No	285	11	3.9	
Occupational contact with animals between 2009 and 2014				0.03
Yes, as veterinarian or veterinarian student^d^	18	4	22.2	
Yes, as an animal health technician or animal health technician student	27	1	3.7	
Yes, other occupations with regular animal contact^e^	26	3	11.5	
No	245	9	3.7	
Having occupational contact with animals and/or having contact with animals during leisure activities between 2009 and 2014				
*Dog and/or cat*				0.09
Yes, during work and leisure activities (dog owners)	58	6	10.3	
Yes, during leisure activities (dog owners)	257	11	4.3	
*Small ruminant*				0.98
Yes, at least during work activities	16	1	6.3	
Yes, during leisure activities only	150	9	6.0	
No	129	7	5.4	
*Bovine and/or cervids*				0.93
Yes, at least during work activities	24	1	4.2	
Yes, during leisure activities only	93	5	5.4	
No	170	10	5.9	
Having contact with new-born domestic animals between 2009 and 2014				
*Puppies and/or kittens*				0.19
Yes, has witnessed birth^f^	59	6	10.2	
Yes, new-born <1 month old	57	4	7.0	
No	183	7	3.8	
*Kids/lambs and/or calves*^g^				0.52
Yes, has witnessed birth^h^	11	1	9.1	
Yes, new-born <1 month old	20	0	0.0	
No	273	16	5.9	
Hunting activities				0.72
Yes^i^	25	1	4.0	
No	284	16	5.6	
Drinking raw milk				0.38
Yes, cow's milk	19	2	10.5	
No	287	15	5.2	

a Participants with missing values were excluded from the analyses, which includes eight participants who did not complete the questionnaire and a varying number of participants who answered ‘I don't know’ for specific questions.

b Likelihood ratio test *P* value.

c For this variable we refer to the year of birth up until the blood collection (so birth up to 2014). In this variable, the category ‘Any type of ruminant farm’ includes cattle, sheep or goat farms.

d Of the 18 participants of this category, 17 are veterinarians and one is a veterinary student.

e Other occupations with regular animal contact mainly includes working in a veterinary clinic, zoo or animal shelter (excluding veterinarians and animal health technicians), on a farm or in a slaughterhouse.

f Of the 59 participants who witnessed the birth of puppies or kittens, 41 were present in the room during birth and 18 were present in the room after birth. Also, 16 had no contact with the birth material, 25 had contact at least once without any protection, 12 had contact with gloves, and six had contact with gloves and a mask.

g The category ‘Yes, has witnessed birth’ was combined with ‘Yes, new-born <1 month old’ for analysis due to lack of model convergence secondary to a category with no seropositive participants.

h Of the 11 participants who witnessed the birth of ruminants, ten were present during birth and one after birth. Also, four were never in contact with birth materials, six were in contact at least once without any protection and one had contact with birth materials with gloves.

i Of the 25 participants taking part in hunting activities, 12 butcher the animal they hunt. The main hunted species are small mammals, birds and cervids.

Participants having lived or worked on a small ruminant farm had 5.4 (95% CI: 1.6–17.7) times the prevalence odds of seropositivity than participants who had not. Veterinarians and veterinary students had 6.2 (95% CI: 1.6–24.0) times the prevalence odds of seropositivity compared to participants who did not work in contact with animals, while the prevalence odds of other occupations with regular animal contact was not statistically significantly higher (Table 6). In the first alternative model, living or working on a bovine farm during a lifetime resulted in a non-statistically significant POR (2.1; 95% CI: 0.7–6.0). In the second alternative model, the POR between living or working on any type of ruminant farm during a lifetime and seropositivity to *C. burnetii* was not statistically significant (POR = 2.5; 95% CI: 0.9–7.0).

**Table 6.** Final multivariable logistic regression model of dog owners' potential risk factors for *C. burnetii* seropositivity in five administrative regions of Québec, Canada, 2014 (*N* = 316)

Variable and categories	PORs		
	Estimate	95% CI^a^	*P* value^a^
Having lived or worked on a small ruminant farm during a lifetime			
Yes	5.4	1.6–17.7	0.006
No	ref.		
Occupational contact with animals between 2009 and 2014			
Yes, as a veterinarian or veterinarian student	6.2	1.6–24.0	0.008
Yes, other occupations with regular animal contact^b^	1.2	0.3–4.7	0.77
No	ref.		

a Wald test 95% CIs and *P* value.

b Other occupations with regular animal contact mainly includes working in a veterinary clinic, zoo or animal shelter (including animal health technician and excluding veterinarians), on a farm or in a slaughterhouse.

Univariable models using spatial data showed no statistically significant association, and no multivariable model could be run because the only two variables with a *P* < 0.20 were too correlated to be both included in the model, i.e. ‘distance between the place of residence and the nearest ruminant farm’ and ‘distance between the place of residence and the nearest bovine farm’ (Table 7).

**Table 7.** Descriptive statistics of participants' living characteristics obtained via spatial analysis from the personal information collected via a questionnaire completed in 2014 and *P* value from univariable logistic regression modelling the seropositivity to *C. burnetii* in five administrative regions of Québec, Canada (*n* = 360) with a sub-analysis for the participants who did not move between 2004 and 2014 (*n* = 170)

Living characteristics of participants	All participants				Participants who did not move^a^			
	Number of participants	Seropositive participants		*P* value^b^	Number of participants	Seropositive participants		*P* value^b^
		*N*	%			*N*	%	
Distance between the place of residence and the nearest farm								
*Any type of ruminant farm*^c^				0.29				0.17
<2 km	129	10	7.8		60	6	10.0	
Between 2 and 5 km	114	4	3.5		58	3	5.2	
>5 km	117	5	4.3		52	1	1.9	
*Bovine farm*				0.20				0.16
<2 km	121	10	8.3		59	6	10.2	
Between 2 and 5 km	119	4	3.4		59	3	5.1	
>5 km	120	5	4.2		52	1	1.9	
*Small ruminant farm*				0.90				0.85
<2 km	50	2	4.0		24	2	8.3	
Between 2 and 5 km	150	8	5.3		78	4	5.1	
>5 km	160	9	5.6		68	4	5.9	
Number of farms in a 5 km radius around the place of residence								
*Any type of ruminant farm*^c^				0.44				0.24
>10	121	9	7.4		56	5	8.9	
⩽10	122	5	4.1		62	4	6.5	
None	117	5	4.3		52	1	1.9	
*Bovine farm*				0.35				0.24
>8	115	9	7.8		56	5	8.9	
⩽8	125	5	4.0		62	4	6.5	
None	120	5	4.2		52	1	1.9	
*Small ruminant farm*				0.87				0.35
>2	86	5	5.8		39	4	10.3	
⩽2	114	5	4.4		63	2	3.2	
None	160	9	5.6		68	4	5.9	
Number of animals in a 5 km radius around the place of residence								
*Any type of ruminants*^d^				0.44				0.26
>715	121	9	7.4		58	5	8.6	
⩽715	122	5	4.1		60	4	6.7	
None	117	5	4.3		52	1	1.9	
*Bovine*				0.43				0.25
>475	120	9	7.5		57	5	8.8	
⩽475	120	5	4.2		61	4	6.6	
None	120	5	4.2		52	1	1.9	
*Small ruminants*				0.32				0.40
>105	125	4	3.2		61	2	3.3	
⩽105	75	6	8.0		41	4	9.8	
None	160	9	5.6		68	4	5.9	

a The sub-analysis only includes the participants who did not move for 10 years before blood collection (so between 2004 and 2014).

b Likelihood ratio test *P* value.

c The category ‘Any type of ruminant farms’ includes cattle, sheep or goat farms.

d The category ‘Any type of ruminants’ includes cattle, sheep or goats.

### Spatial distribution

**Fig. 3.**
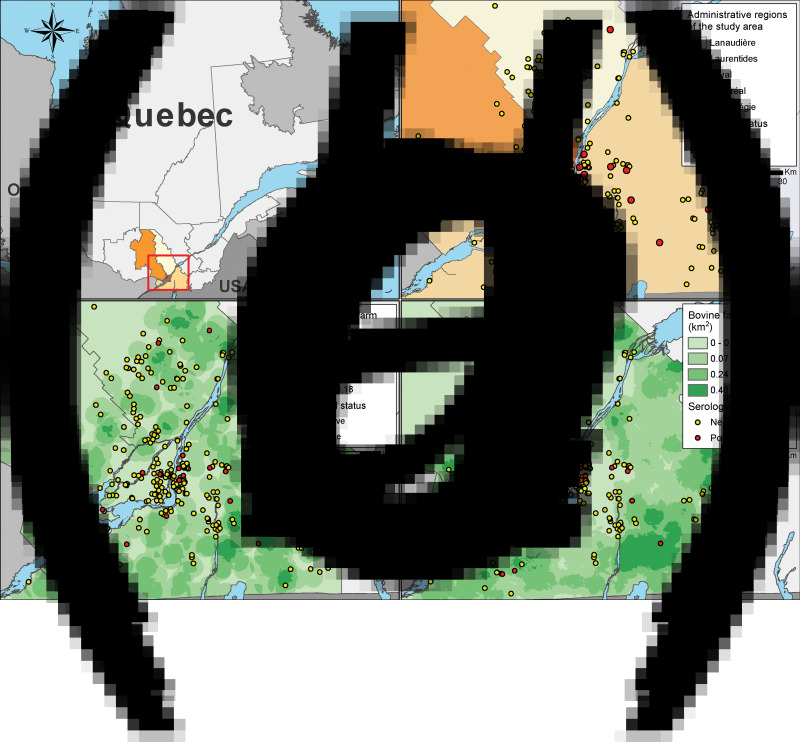
Study area, geographical distribution of human participants and distribution of ruminant farm density in 2014 in Québec, Canada, for a *C. burnetii* serological study. (A) Study area: southwestern portion of the province of Québec, Canada. (B) Geographical distribution of the participants (360) according to their *C. burnetii* serological status in the five administrative regions studied (qualitatively identified). (C) Geographical distribution of the small ruminant farm density (km^2^) calculated in ArcGIS using a point density in a 5 km radius around the participants' place of residence. (D) Geographical distribution of the bovine farm density (km^2^) calculated in ArcGIS using a point density in a 5 km radius around the participants' place of residence.

The geographical distribution of the participants according to their serological results, as well as the bovine and the small ruminant farm densities in the five studied administrative regions, are mapped in Figure 3. No statistically significant clusters were found using the Kulldorff spatial scan test including all participants (*n* = 360, seropositive participants = 19, all *P* ⩾ 0.6) or only the participants that reported having no occupational contact with animals (*n* = 245, seropositive participants = 9, *P* > 0.4).

## Discussion

This exploratory study investigated *C. burnetii* seropositivity in an adult population of dog owners in five administrative regions of southwestern Québec. This study adds to the limited available knowledge regarding factors associated with human seropositivity to *C. burnetii* in endemic regions. The variation of ruminant farm densities by region allowed for an investigation of the impact of living in proximity to ruminant farms as well as living in the areas of various densities of ruminant populations, in addition to individual characteristics of exposure to *C. burnetii*.

Both the IFA and the ELISA are serological tests used for Q fever diagnosis [26]. However, no test and threshold has been identified as the preferred method for serosurveys on *C. burnetii*, but some have recommended a screening by an ELISA and confirmation of the negative or positive results with an IFA to improve the sensitivity or specificity, respectively [27, 28]. In our study, a low antibody threshold was used during screening with the ELISA to maximise sensitivity. Confirmation of the ELISA-equivocal and ELISA-positive results was then performed using an IFA. Cross-reactions with *Legionella pneumophila* have been reported with the Panbio IgG ELISA kit [24]. The low percentage of IFA-positive results among the ELISA-equivocal sera in addition to the IFA-negative results obtained from all samples with a rounded Panbio index value of 0.8 suggests that the method used detected most of the samples that would have been positive if an IFA was performed on all samples (Fig. 2). The seroprevalence estimates were not adjusted for the sensitivity and specificity of the diagnostic procedure because no reliable values for the validity of the IFA could be found in the literature since it is considered the reference test for clinical diagnosis [27].

No statistically significant difference was observed between the regions despite an expectation for a higher seroprevalence in the region with highest ruminant farm density, Montérégie. It should be noted that the seroprevalence estimates observed in Laval (11.1%; 95% CI: 0.3–48.2) and Laurentides (0%; 95% CI: 0.0–23.2) regions are based on small sample sizes and should be interpreted with caution. In Laval, the only positive participant detected had been in contact with goats once a year between 2009 and 2014. In the Laurentides, the absence of detection is most likely due to the small sample size rather than the absence of exposure, as cases of Q fever were previously reported in this region [14]. Only one positive participant was detected in Montréal, an urban city. This participant reported occasionally doing chores on a stable as the only potential risk factor of exposure to *C. burnetii*. Contact with horses has been identified as a possible risk factor for *C. burnetii* infection in humans [29] but stray cats can also be found in stables and could be a potential source of transmission to humans.

When the two regions with small sample sizes are excluded, our seroprevalence estimates from the three remaining regions are similar to what has been reported (3.1%; 95% CI: 2.1–4.3) in the general adult (⩾20 years of age) population of the United States, using the same combination of tests used in this study [30]. They are also close to the 2.4% of seropositive participants reported prior to the 2007 outbreak in the Netherlands, the largest Q fever outbreak ever documented, following a nationwide seroprevalence survey using a similar serological testing method as this study [31]. The seroprevalences estimated in our study and the ones reported by other studies among the human and ruminant populations of Québec might reflect a situation of endemicity in areas with limited livestock raising [15, 32].

The Q fever questionnaire focused on questions less likely to be affected by poor recall since it was completed in 2018, 4 years after sera had been collected. That said, errors in recall are still possible for some of the questions such as having had contact with ruminants during leisure activities in the past. However, since the questionnaire was completed before the serological results were available, and participants reported never having been diagnosed with a *C. burnetii* infection, if a misclassification bias is present, it would be non-differential, and would most likely lead to an under-estimation of the true association.

Studies have investigated the association between seropositivity to *C. burnetii* and living or working on a ruminant farm (all species combined). In Northern Ireland, odds of seropositivity were higher among farmers in comparison with non-farmers (OR = 5.3; 95% CI: 3.4–8.5) [33]. In the Netherlands, before the 2007–2010 Q fever outbreak, seropositivity was associated with keeping ruminants with other farm animals (OR = 8.2; 95% CI: 3.3–20.8) and without other farm animals (OR = 3.8; 95% CI: 1.1–13.1) [31]. In this study, the potential risk factor of living or working on a farm was also investigated separately for small ruminants and bovine. The multivariable analysis showed that having lived or worked on a small ruminant farm during a lifetime was positively associated with *C. burnetii* seropositivity (POR = 5.4; 95% CI: 1.6–17.7). On the other hand, having lived or worked on a bovine farm during a lifetime was not statistically significant. These findings could partly be explained by the higher prevalence of *C. burnetii* positivity reported in ovine herds (70.8%) of Québec compared to the one reported in bovine herds (44.6%) of the province [32]. Another hypothesis relates to differences in reproduction management; unlike dairy cattle herds, parturitions in small ruminant herds are usually grouped in batches. Additionally, *C. burnetii* infections cause abortions more frequently in small ruminants than in cattle, and they can occur as a herd-level epidemic in small ruminants compared to only sporadic events in cattle [26, 34]. Small ruminant farm workers might therefore be periodically exposed to high levels of *C. burnetii*, hence their increased risk compared to cattle farm workers. However, studies have identified cattle farmers and cattle farm residents as a group at risk of *C. burnetii* infection, as high seroprevalences were observed among these individuals [10, 12, 35]. Even though living or working on a bovine farm was not found to be a risk of *C. burnetii* seropositivity in this study, the risk could have gone undetected because it is likely weaker as supported by our descriptive analyses. If so, the risk should not be dismissed since the fraction of seropositive people in the population attributable to cattle exposure could be significant as our study area included 5.3 times more cattle farms than small ruminant farms.

It is well established that veterinarians and veterinary students are at greater risk of *C. burnetii* infection [1, 26], and our study supports this conclusion. Despite veterinarians being aware of the risk of transmission of this zoonotic bacterium when in contact with animals, preventive measures previously identified such as wearing a mask and protective cloths are not consistently applied [36]. Indeed, we observed in our study that among veterinarians reporting assisting parturition, only a minority (3/14) reported wearing a mask and gloves. The reasons for non-compliance of veterinarians when at risk should be investigated.

No statistically significant differences were observed between participants working in contact with animals, excluding veterinarians and veterinary students, and those who did not work with animals. This is likely because close to a third (17/53) of the participants who reported working with animals were actually working in an occupation with minimal or occasional contact with animals, such as working as a receptionist in a veterinary clinic.

No associations between living in proximity to ruminant farms and *C. burnetii* seropositivity were found to be statistically significant. However, the descriptive statistics suggested that people living close (2 km radius) to a bovine farm are possibly at higher risk than those living further away. Ruminants are a possible source of *C. burnetii* transmission to humans in Québec as coxiellosis is endemic in ruminant herds in this province. A systematic review on Q fever outbreaks reported that small ruminants, but not cattle, were identified as the likely source for all the outbreaks, and that living in proximity to those farms was a significant risk factor for infection [4]. A study of the substantial Q fever outbreak in the Netherlands identified living within 2 km of a positive goat farm as a high risk factor for seropositivity (relative risk 31.1; 95% CI: 16.4–59.1) [21]. Additionally, living within 1 km of a farm with more than 50 goats was significantly associated with *C. burnetii* seropositivity (prevalence ratio 1.9; 95% CI: 1.2–3.0) [37]. In the Netherlands, the reported ruminant densities of the most affected region were approximately 42 goats or sheep/km^2^ and 129 cattle/km^2^ [3] while the densities observed in this study were much lower with maximums of 0.04 goats or sheep/km^2^ and 0.25 cattle/km^2^ (Table 1). This density difference could explain why the risk associated with living close to a small ruminant farm is higher in the Netherlands compared to these regions of Québec. As previously discussed, the role of cattle in *C. burnetii* transmission to human should be further investigated in Québec since no clear conclusion could be drawn in this study.

The regional seroprevalences show that *C. burnetii* may be of public health concern in southwestern Québec. Q fever is a notifiable disease in this province of Canada, and in the five studied regions, 29 Q fever cases were reported from 2011 to 2014, with Montérégie being the region with the highest number of cases, which is consistent with our results as the highest number of seropositive participants were detected in this region.

In this study, higher seropositivity was identified in veterinarians and people living or working on small ruminant farms. In Québec, public health messaging is designed to raise awareness among goat and sheep farmers and people working with small ruminants. It also educates people with confirmed Q fever cases so they can prevent new infections. Despite government interventions and the education provided to veterinarians, the risk to these individuals is still high. Vaccination of people at a high risk of infection might be potentially considered, as vaccination is currently used in Australia for certain occupational groups [38]. However, human vaccination can cause hypersensitivity reactions in people who have been previously exposed to this zoonotic agent, and no human vaccine against Q fever is readily available in Canada [1, 39]. Then, if the objective is to prevent endemic or epidemic Q fever cases in the general population or in high-risk populations, it could be more effective to intervene at the animal level. The vaccination of small ruminant herds may result in a decrease in bacterial excretion and a reduction in environmental contamination and human infection [40]. An economic study reported that the cost of a small ruminant vaccination programme is relatively low compared to the public health costs that may follow a large Q fever outbreak like the one that occurred in the Netherlands between 2007 and 2010 [41]. The cost–benefit ratio of ruminant vaccination should not only consider the advantages on the prevention of Q fever outbreaks, but also its potential impacts on the reduction of endemic cases in both humans and animals.

### Limitations

Participants of this study were not randomly selected; they were selected from a convenience pool of dog owners attending veterinary clinics and hospitals. Since the recruitment of participants was conducted in veterinary clinics and because veterinarians were allowed to participate, veterinarians might be overrepresented in this study. Indeed, veterinarians represent about 5% of the participants, while the estimated proportion of veterinarians in the population of the studied regions is about 0.05% [42]. Therefore, we believe that the seroprevalence estimates from this study might be overestimated, so inferences to the human population of southwestern Québec should be made cautiously. Additionally, according to a survey conducted in 2013 in the province of Québec, only 24% of Québec households own at least one dog [43]. There is no evidence that owning a dog increases the risk of *C. burnetii* infection, but dog owners might be more inclined to be in contact with other animals and spend time outdoors.

A slightly higher proportion of seropositive participants was observed in the subset who completed the Q fever questionnaire (5.4%) than overall (4.2%). Apart from random variation, we do not have a clear explanation for this finding. Among all participants, 83% of veterinary and 88% of non-veterinary participants completed this questionnaire.

This study was a secondary analysis and therefore had a fixed sample size of 485 participants. As a result, the power and precision of the seroprevalence estimates and the risk factor analysis could not be optimised by increasing the sample size. This limitation mostly affected the precision of the prevalence estimates of two regions, Laurentides and Laval, and possibly the ability to detect weaker associations.

## Conclusion

The results obtained in this study show that exposure to *C. burnetii* occurs in the human population in at least four of the five studied administrative regions of the province of Québec, Canada. Living or working on a small ruminant farm and being a veterinarian were the two factors found to be associated with *C. burnetii* seropositivity in this study. Ruminant farm proximity or density were not found to be significantly associated with seropositivity in this study, but studies with an adequate sample size would improve the understanding of environmental risk factors in an endemic setting to complement prevention and control strategies already in place. The implementation of a small ruminant vaccination programme could be an effective approach to decrease occupational risk and to reduce the general population risk of exposure to *C. burnetii*.

## Data Availability

The anonymised datasets used during the current study are available from the corresponding author upon a reasonable request.
